# Peripheral Leukocyte Migration in Ferrets in Response to Infection with Seasonal Influenza Virus

**DOI:** 10.1371/journal.pone.0157903

**Published:** 2016-06-17

**Authors:** Nedzad Music, Adrian J. Reber, Jin Hyang Kim, Ian A. York

**Affiliations:** 1 Influenza Division, Centers for Disease Control and Prevention, Atlanta, Georgia, United States of America; 2 Battelle Memorial Institute, Atlanta, Georgia, United States of America; University of Alabama at Birmingham, UNITED STATES

## Abstract

In order to better understand inflammation associated with influenza virus infection, we measured cell trafficking, via flow cytometry, to various tissues in the ferret model following infection with an A(H3N2) human seasonal influenza virus (A/Perth/16/2009). Changes in immune cells were observed in the blood, bronchoalveolar lavage fluid, and spleen, as well as lymph nodes associated with the site of infection or distant from the respiratory system. Nevertheless clinical symptoms were mild, with circulating leukocytes exhibiting rapid, dynamic, and profound changes in response to infection. Each of the biological compartments examined responded differently to influenza infection. Two days after infection, when infected ferrets showed peak fever, a marked, transient lymphopenia and granulocytosis were apparent in all infected animals. Both draining and distal lymph nodes demonstrated significant accumulation of T cells, B cells, and granulocytes at days 2 and 5 post-infection. CD8+ T cells significantly increased in spleen at days 2 and 5 post-infection; CD4+ T cells, B cells and granulocytes significantly increased at day 5. We interpret our findings as showing that lymphocytes exit the peripheral blood and differentially home to lymph nodes and tissues based on cell type and proximity to the site of infection. Monitoring leukocyte homing and trafficking will aid in providing a more detailed view of the inflammatory impact of influenza virus infection.

## Introduction

Influenza A viruses are common human respiratory pathogens causing significant morbidity and mortality worldwide [1–3]. Seasonal human influenza viruses, including A(H3N2) and 2009 pandemic A(H1N1)pdm09, usually target the upper respiratory tract. In most cases, these upper respiratory tract infections are cleared and the individual develops immunity to the specific strain of virus, although antigenic variants may escape this immunity through antigenic drift to infect the same person in subsequent years. The disease is occasionally severe, either when influenza infection predisposes patients to secondary infection with bacteria which rarely cause serious infections alone, or when the influenza virus spreads to the lower respiratory tract and virus alone leads to localized or systemic inflammation and severe disease [4–6].

Inflammation and leukocyte trafficking go hand in hand [7]. Indeed, much of the recent anti-inflammatory drug development has focused on trafficking molecules and control of leukocyte trafficking as a means of dampening inflammation [7, 8]. Inflammation associated with influenza infection has been extensively studied in mice [9–11]. In humans, however, it is less well understood. Small animal models for influenza infection include mice, guinea pigs, and ferrets. In contrast to mice and guinea pigs, human and avian influenza viruses replicate efficiently in the respiratory tract of ferrets without prior adaptation, and in general the course of infection in ferrets is very similar to that in humans. The ferret is therefore considered the most suitable small animal model for influenza virus infection and vaccine protection studies [12–14]. One major disadvantage of the ferret model of influenza virus infection and immunity, however, is the paucity of ferret-specific reagents available for analysis of the response. In particular, identification of leukocyte subsets has been difficult, making it challenging to analyze the inflammatory response to infection. Migration of immune cells between compartments in response to inflammatory mediators is critical for establishing effective T and B cell mediated immune responses, and creating adaptive immune memory as protection against further infection [15–17]. We recently adapted and extended previous findings in order to track ferret peripheral blood leukocyte (PBL) subsets on a daily basis following seasonal influenza virus infection [18]. We found that, even though clinical symptoms were mild as previously reported [19–21], leukocyte subsets in peripheral blood showed rapid, dynamic, and profound changes in response to infection, with a marked transient lymphopenia and moderate granulocytosis early in the infection followed by a gradual recovery to normal leukocyte values over about 7 days [18]. The mechanisms causing changes in peripheral leukocyte numbers were not clear. Since we only examined peripheral blood, migration into the respiratory tract [21] or lymphoid tissues seemed likely to account for at least part of the loss of lymphocytes. To gain a better understanding of inflammation associated with influenza infection and protection afforded by cell-mediated immunity, we conducted an analysis of leukocyte subsets in tissues, as well as peripheral blood, following influenza infection using the ferret model.

## Methods and Materials

### Ethics Statement

This study was carried out in strict accordance with the Animal Welfare Act regulations of the United States Department of Agriculture and Public Health Service Policy on Humane Care and Use of Laboratory Animals administered by the National Institutes of Health. All animal research was conducted under a Centers for Disease Control and Prevention’s Institutional Animal Care and Use Committee approved protocol. Animal welfare was monitored on a daily basis, and all animal handling was performed under light anesthesia (as described below); all efforts were made to minimize suffering. Humane endpoints for this study included the presentation of body weight loss exceeding 20% (relative to weight at challenge), indications of neurological symptoms, or a clinical score of 3 in any category based on the system designed by Reuman et al [22]. However, none of the animals in this study met those criteria.

### Virus

Virus stocks for influenza A(H3N2), A/Perth/16/09 (Perth/16), were propagated in the allantoic cavity of 10 day-old fertile white embryonated chicken eggs (Hy-line, Mansfield, GA) at 34°C for 48h. Virus containing allantoic fluid was harvested, aliquoted and frozen at −80°C until usage in experiments. Stocks were titrated in a standard plaque assay and expressed as plaque forming units (pfu) [23] using Madin-Darby Canine Kidney (MDCK) cells.

### Ferrets, samples, and viral challenge

Male Fitch ferrets, approximately 6 months of age (Triple F Farms, Sayre, PA), serologically negative by hemagglutination inhibition (HI) assay for currently circulating human influenza H1, H3 and type B viruses, were used in this study. Prior to initiation of the studies, all ferrets were evaluated by a licensed veterinarian and determined to be in good health and free from malformations and signs of clinical disease. During the 14 day quarantine period, animals were randomized by weight into groups. Body temperatures were measured using an implantable subcutaneous temperature transponder (BioMedic Data Systems, Inc., Seaford, DE). Baseline weights and temperatures were obtained for three consecutive days prior to challenge and on day 0 (the day of challenge).

**Fig 1:**
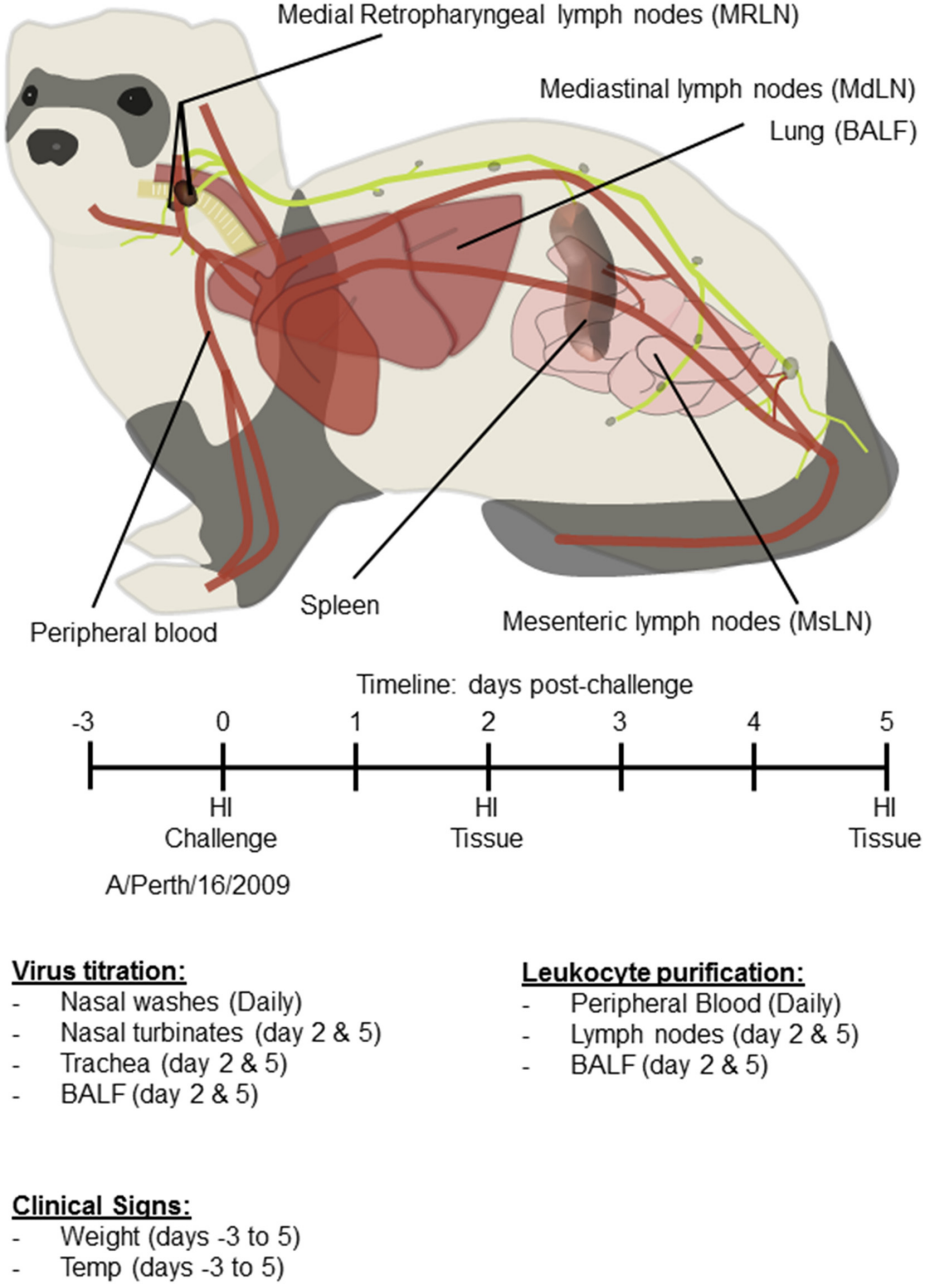
Experimental protocol. Male Fitch ferrets (Mustela putorius furo), approximately 6 months of age were used in this study. Groups of 8 ferrets were infected intranasally with 1 x 10^6^ pfu of A/Perth/16/09, or groups of 4 to 6 ferrets were mock infected. Baseline weights and temperatures were obtained for the three consecutive days prior to challenge and on day 0 (the day of challenge). Following challenge, ferrets were monitored for change in body weight and temperature as well as clinical signs of illness on a daily basis for 5 days. Blood samples were collected for Flow Cytometry (days 0–5) and serum collected (days 0, 2 and 5) to asses antibody responses (HI assays). All ferrets were euthanized on day 2 (4 Perth/16-infected ferrets) or day 5 post-challenge (4 Perth/16-infected and all mock-infected ferrets). Viral titers were determined from nasal washes (days 0–5) nasal turbinates (days 2 and 5), BALF (days 2 and 5) and trachea (upper and lower regions; days 2 and 5). Leukocyte purification was performed from peripheral blood (days 0–5), from lymph nodes (days 2 and 5) and from BALF (days 2 and 5).

The experimental design is outlined in Fig 1. Groups of 8 ferrets were infected intranasally with 1 x 10^6^ pfu of Perth/16 diluted in 0.5 ml of sterile phosphate-buffered saline (PBS), or groups of 4 to 6 ferrets were mock infected with 0.5 ml of a similar dilution of sterile egg allantoic fluid. The experiment was repeated three times with a total of 24 virus infected and 14 mock infected ferrets; 1 infected ferret was discovered to have advanced lymphoma upon necropsy and all data from that ferret was removed from analysis.

Following viral challenge, ferrets were monitored for changes in body weight and temperature as well as clinical signs of illness (sneezing, lethargy, nasal discharge, diarrhea and neurological dysfunction) on a daily basis until the end of the study. Blood samples were collected daily as previously described [18]. Briefly, animals were anesthetized with ketamine/xylazine, and blood samples of 200–250 μl were taken from the cranial vena cava [24] from day 0 to the end of the experiment. For virus titration, nasal washes were collected daily, as described previously [14].

All ferrets were euthanized either on day 2 (4 Perth/16 infected ferrets) or day 5 post-challenge (4 Perth/16 infected and all mock infected ferrets). These days were chosen since PBL changes were at their maximum levels on day 2, followed by a second smaller peak on day 5 [18]. Selected tissues were collected for screening of immune cell migration in response to influenza infection. In addition, viral titers were determined in tissues from euthanized ferrets. Nasal turbinates, bronchoalveolar lavage fluids (BALF) and trachea (upper and lower regions) were collected at days 2 and 5 post-challenge (Fig 1). Nasal turbinates and trachea were harvested, weighed, and homogenized in 1ml of cold PBS. The material was clarified by centrifugation (700×*g* for 10 min, at 4°C) and the supernatant used for virus quantification. Virus titers were determined by a plaque assay in MDCK cells and expressed as log10 pfu in 1 ml of nasal wash or BALF; or 1 g of tissue sample.

### Purification of cells from whole blood, BALF, Lymph Nodes and Spleen

Peripheral blood leukocytes (PBLs) were purified as previously described [18] by hypotonic lysis of red blood cells using erythrocyte lysing solution (0.15 M NH_4_Cl, 10 mM KHCO_3_, and 1mM EDTA pH 7.3).

BALF was collected after euthanasia as previously described [25, 26] with some modifications. The chest cavity was opened to expose the lungs and an incision was made to expose the trachea. The trachea was cut approximately in the middle of its length (~ 5 cm before the bifurcation of the bronchi) and clamped, after which the entire respiratory tract was carefully removed with the heart. Sterile PBS (5 ml) containing BSA was infused slowly down the trachea into the lung. The lungs were gently massaged and inverted, and fluid was allowed to drip back through the trachea and collected. Approximately 3 ml of the fluid was recovered and stored on ice. The cells from lavage fluid were collected following centrifugation (315×*g* for 10 min, at 4°C). The absolute number of leukocytes from BALF were estimated by dividing the number of cells collected from the BALF by the volume of liquid recovered, and multiplying by the volume of liquid delivered (5 ml).

Medial retropharyngeal lymph nodes (MRLN), mediastinal lymph nodes (MdLN), mesenteric lymph nodes (MsLN), and spleens were removed from each ferret immediately after euthanasia. Single-cell suspensions from lymph nodes were made by gently rubbing partially minced tissues through sterile stainless steel mesh (size 40 micron, Sigma Chemical Co., St Louis MO) using a 10 ml syringe plunger. Single-cell suspensions from spleens were made by gently grinding them between two frosted glass slides. Splenocyte cell pellets were suspended in erythrocyte lysing solution (0.15 M NH_4_Cl, 10 mM KHCO_3_, and 1mM EDTA pH 7.3) for 6 min at room temperature and then pelleted again by centrifugation. All cell suspensions were collected, filtered through 70 μm cell strainers (BD Bioscience, San Jose, CA), and pelleted by centrifugation (315×*g* for 10 min at 4°C). Cell pellets were washed twice, counted, and suspended in flow buffer for flow cytometry staining.

### Flow Cytometry

Flow cytometry assays were performed as previously described [18]. Briefly, cells were blocked with Fc blocking antibody (Clone 2.4G2, Cat Nu 553142, BD Biosciences, San Jose, CA) and stained with monoclonal antibodies recognizing ferret CD4 (clone 02 –PE, Sino Biological Inc., Beijing, China), or cross-reacting with ferret CD8 (clone OKT8 –eFluor 450, eBioscience, San Diego, CA), CD11b (clone M1/70 –FITC, eBioscience), anti-MHC class II (clone CAT82A –unconjugated, Kingfisher Biotech, St. Paul, MN), CD3 (clone PC3/188A –FITC and AlexaFluor 647, Santa Cruz Biotechnology, Santa Cruz, CA), and CD79a (clone HM47 –PerCP-Cy5.5, eBioscience). The anti-MHC class II antibody was biotinylated (cat# 130-093-385, Miltenyi Biotec, San Diego, CA) prior to use and detected with streptavidin-eFluor 450 (eBioscience). Unstained cells and isotype controls (eBioscience, San Diego, CA) were included for all antibodies. Data were acquired using a FACSCanto II flow cytometer (BD Bioscience, San Jose, CA), and analyzed using FlowJo software (Tree Star, Ashland, OR).

### Serology

To assess antibody responses, serum was collected prior to infection, on the day of viral challenge, and 2 and 5 days post-challenge. Sera were stored at -20°C until use. HI assays were performed as previously described [27]. HI titers against influenza A/California/07/2009 (A(H1N1)pdm09), A/Perth/16/2009, and B/Brisbane/60/2008 viruses were assessed and expressed as the reciprocal of the highest dilution of the samples inhibiting hemagglutination.

### Statistical analysis

The experiments were repeated three times, including a total of 23 influenza infected and 14 mock infected ferrets. Sampling performed on days 2 and 5 were analyzed using Dunnett’s multiple mean comparison with all comparisons performed against mock infected animals (Prism; GraphPad Software, Inc., La Jolla, CA). Time-course analyses were calculated using a linear mixed model with repeated measures using SAS software (SAS Institute Inc., Cary, NC). Post-challenge data were analyzed both as separate experiments and as merged data, with very similar results; figures shown here are from merged data.

## Results

### Clinical signs after viral challenge

All ferrets were seronegative for influenza viruses before infection, and animals remained seronegative by HI to the challenge virus, as well as to currently circulating B, H1 and H3 strains, throughout the 5-day study (data not shown).

**Fig 2:**
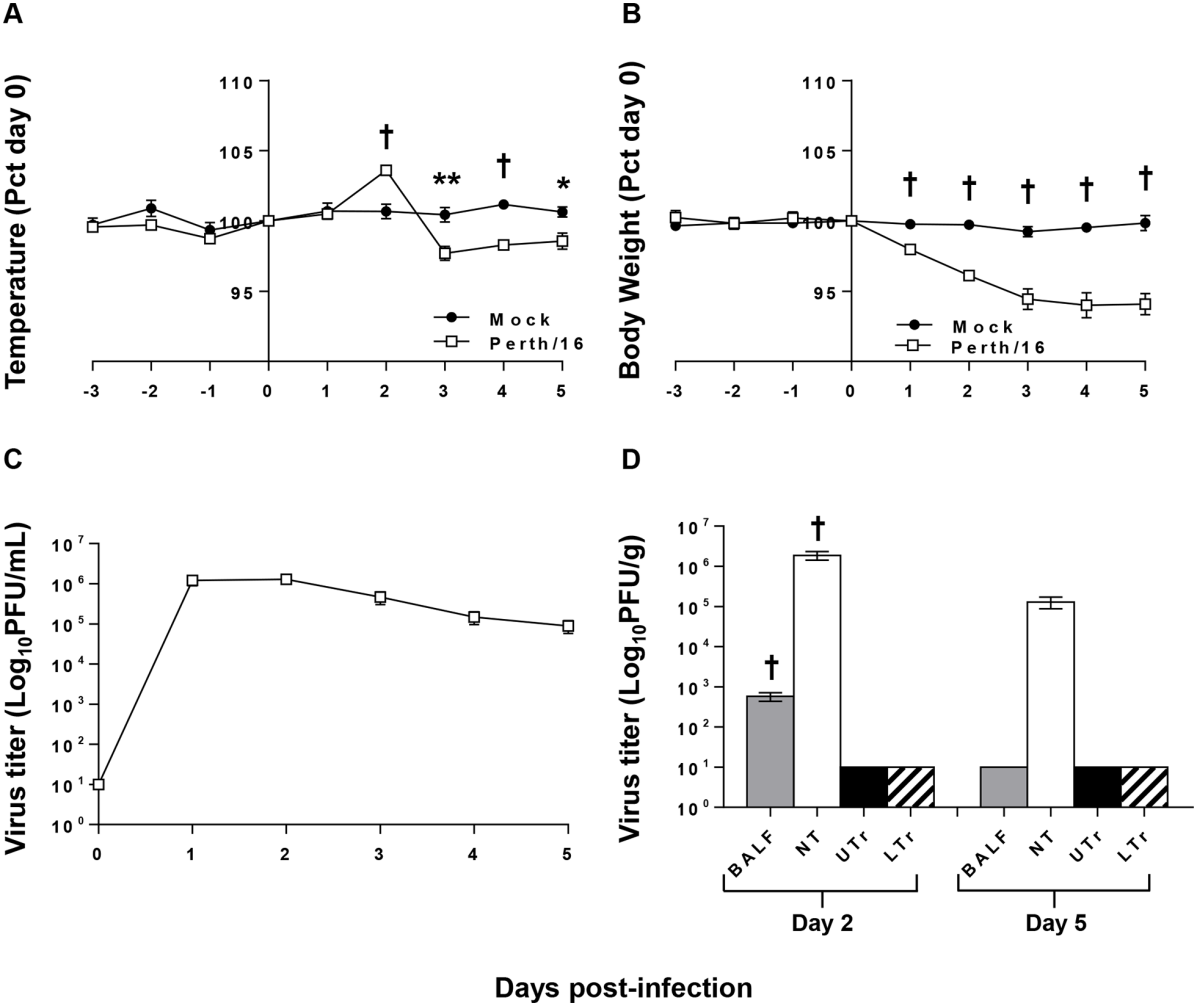
Clinical responses to infection. To measure morbidity the temperature (A) and body weight (B) readings of infected and control ferrets were recorded daily (days -3 to 5). Data shown are normalized to the individual animals’ weight or temperature on the day of challenge (day 0), and group averages are reported. “Mock” ferrets were infected intranasally with sterile egg allantoic fluid; “Perth/16” ferrets infected intranasally with 1 x 10^6^ pfu of A/Perth/16/09. The nasal cavities of all ferrets were washed daily with 1ml of PBS on days 0–5; and viral titers in the nasal washes (C) were determined. In addition, viral titers were determined in tissues (D); BALF, nasal turbinates (NT), upper trachea (UTr), and lower trachea (LTr). A p value of 0.05 was used as the cutoff for statistical significance (* p ≤ 0.05; ** p ≤ 0.01; † p ≤ 0.001). Error bars represent SEM.

Ferrets mock-infected with diluted, sterile allantoic fluid did not show clinical signs, while those infected with Perth/16 all showed mild clinical signs of influenza infection (Fig 2A and 2B). Perth/16-infected ferrets exhibited a spike in body temperature two days post-challenge, followed by a reduction in temperature on days 3–5 post-challenge, showing significantly lower body temperature than non-infected ferrets (Fig 2A). Perth/16-infected ferrets lost between 2.5% and 10.3% of body weight (mean maximum weight loss, 6%) (Fig 2B). As previously shown [18], non-infected animals maintained their body weight and temperature (Fig 2A and 2B) despite daily sedation and blood collection.

Virus replication in the upper respiratory tract was determined by titrating nasal washes. Peak viral shedding was observed on day 1 or 2 post-challenge (Fig 2C), and ferrets continued to shed virus throughout the 5-day experiment, although titers were much lower by five days post-infection (Fig 2C). In addition, we measured viral titers in tissues from the upper respiratory tract (nasal turbinates and upper part of trachea) and from the lower respiratory tract (BALF and lower part of trachea). Two days post-challenge, 11 of 12 ferrets (91.7%) had detectable virus in BALF, but no virus was detected in BALF by day 5 after challenge (Fig 2D). As described previously [21, 28], seasonal A(H3N2) replicated very effectively in nasal turbinates of ferrets, although by five days post-challenge viral titers in turbinates were substantially reduced (p ≤ 0.001; Fig 2D). No virus was detected in the trachea at any time (Fig 2D).

### Changes in leukocyte subsets after challenge

**Fig 3:**
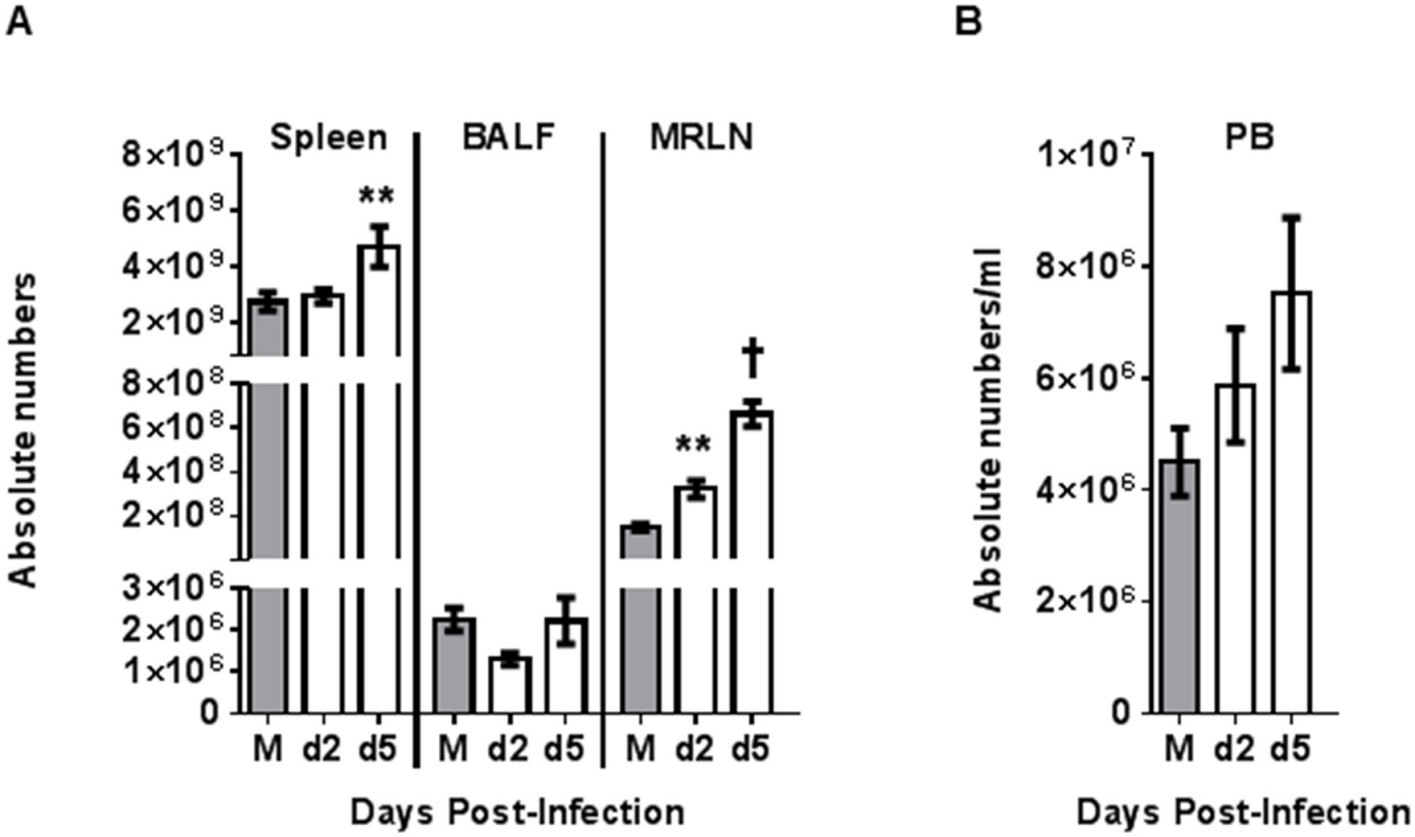
Tissue leukocyte cell counts following infection. For evaluation of cellularity, the total cell numbers were calculated in spleen, MRLN, BALF (A), and in peripheral blood (PB) (B). Group averages are reported. For the mock-infected animals (M), all data are collected on day 5 post-challenge, and for the virus-infected animals on days 2 and 5 post-challenge (d2 and d5). A p value of 0.05 was used as the cutoff for statistical significance (* p ≤ 0.05; ** p ≤ 0.01; † p ≤ 0.001). Error bars represent SEM.

For evaluation of cellularity we calculated the total cell numbers in spleen, MRLN, BALF (Fig 3A), and peripheral blood (Fig 3B). Since the MdLN and MsLN are widely dispersed and not well demarcated, it was impossible to collect total lymphoid material from these regions and total numbers could not be estimated. The total number of cells significantly increased (P≤0.01) in spleen on day 5 post-challenge (Fig 3A). Total cell counts in BALF remained approximately constant over the post-challenge period for all three groups of ferrets, although Perth/16 infected animals euthanized on day 2 post-challenge showed a slight but non-significant decrease in total cell counts (Fig 3A). For the MRLN, total cell numbers increased significantly both on day 2 (P≤0.01) and day 5 (P≤0.001) post-challenge (Fig 3A). PBL counts increased slightly, but non-significantly, on days 2 and 5 post-challenge (Fig 3B).

**Fig 4:**
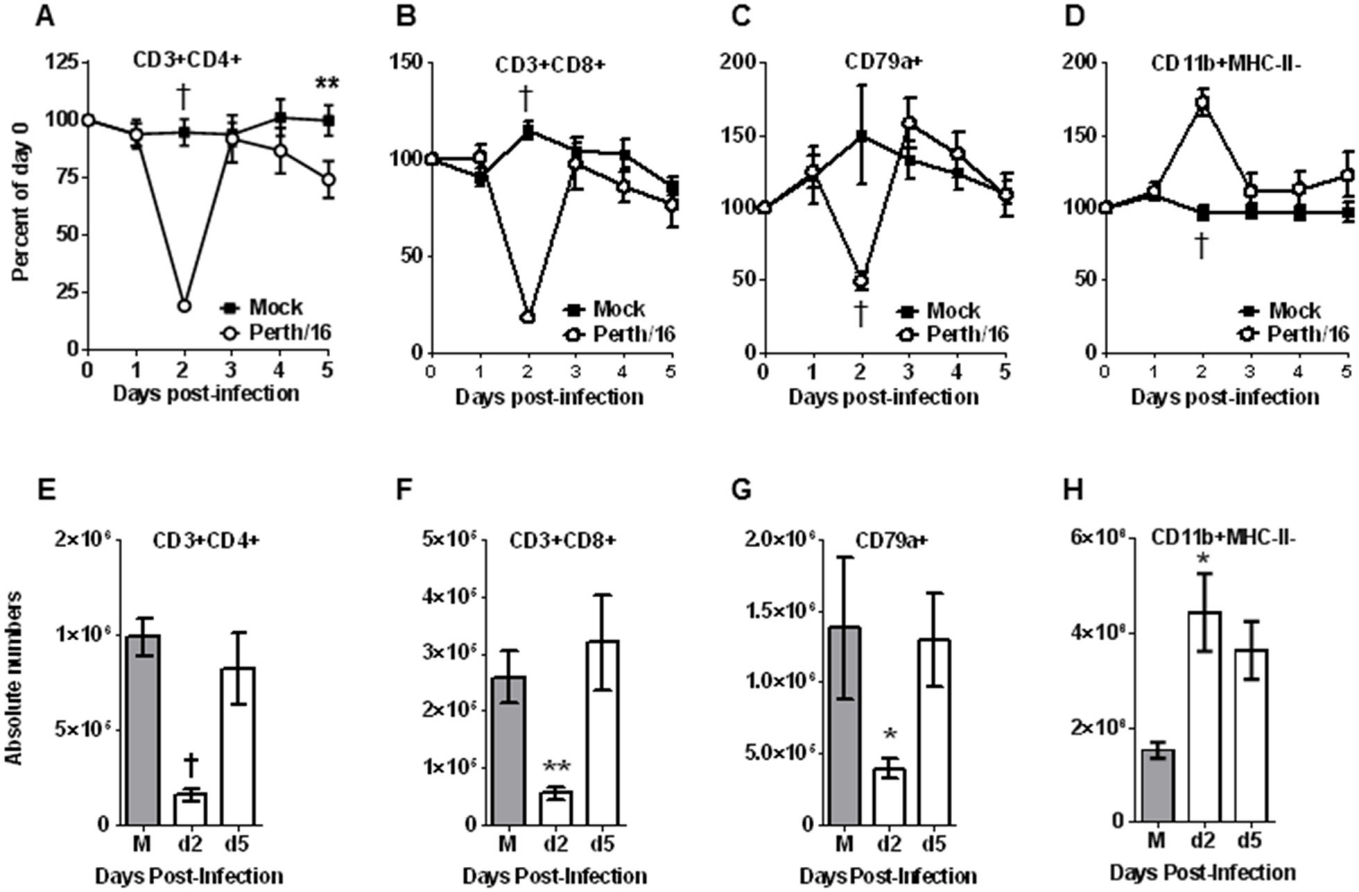
Peripheral blood leukocyte subsets following infection. Ferrets were bled on days 0–5 relative to the day of viral challenge, and cells were stained and analyzed by flow cytometry as described in the text. Percentage and absolute number of T_H_ (CD3+ and CD4+), Tc lymphocytes (CD3+ and CD8+), B cells (CD79a+) and CD11b-positive cells were measured (A-H). For each animal the frequencies were normalized to the ferret’s values on day 0; the Y axis represents percent of values on day 0. Group averages are reported here. “Mock” ferrets were infected intranasally with sterile egg allantoic fluid; “Perth/16” ferrets infected intranasally with 1 x 10^6^ pfu of A/Perth/16/09. For mock animals (M), absolute number of same cell subsets were measured at day 5 post-challenge and for the infected animals at days 2 and 5 post-challenge. A p value of 0.05 was used as the cutoff for statistical significance (* p ≤ 0.05; ** p ≤ 0.01; † p ≤ 0.001). Error bars represent SEM.

In peripheral blood, leukocyte dynamics followed the same pattern as previously described [18]. Two days after infection, when infected ferrets showed peak fever, the percentage of both CD4+ (Fig 4A) and CD8+ (Fig 4B) T cells decreased dramatically in peripheral blood, to about 19% and 18% of pre-challenge levels respectively, with some animals showing a disappearance of as much as 95% of CD8+ T cells on day 2. By day 3, both CD4+ and CD8+ cells returned to nearly baseline (day 0) levels, with a second drop on days 4 and 5 (Fig 4A and 4B). Values for total CD3+ cells are shown in the supplementary data (S1 and S2 Figs). The percentage of B cells (CD79a+) also showed a marked drop 2 days after infection (Fig 4C), to 50–60% of baseline levels, but returned to approximately normal levels after day 2. The percentage of granulocytes (CD11b+; presumably predominately neutrophils) in peripheral blood increased post-infection, with a peak on day 2 post-infection (Fig 4D), and also essentially returned to normal levels after day 2. As previously shown [18], peripheral blood changes reflected both percentages and absolute number of cells (Fig 4E and 4H, S1C and S1D Fig).

**Fig 5:**
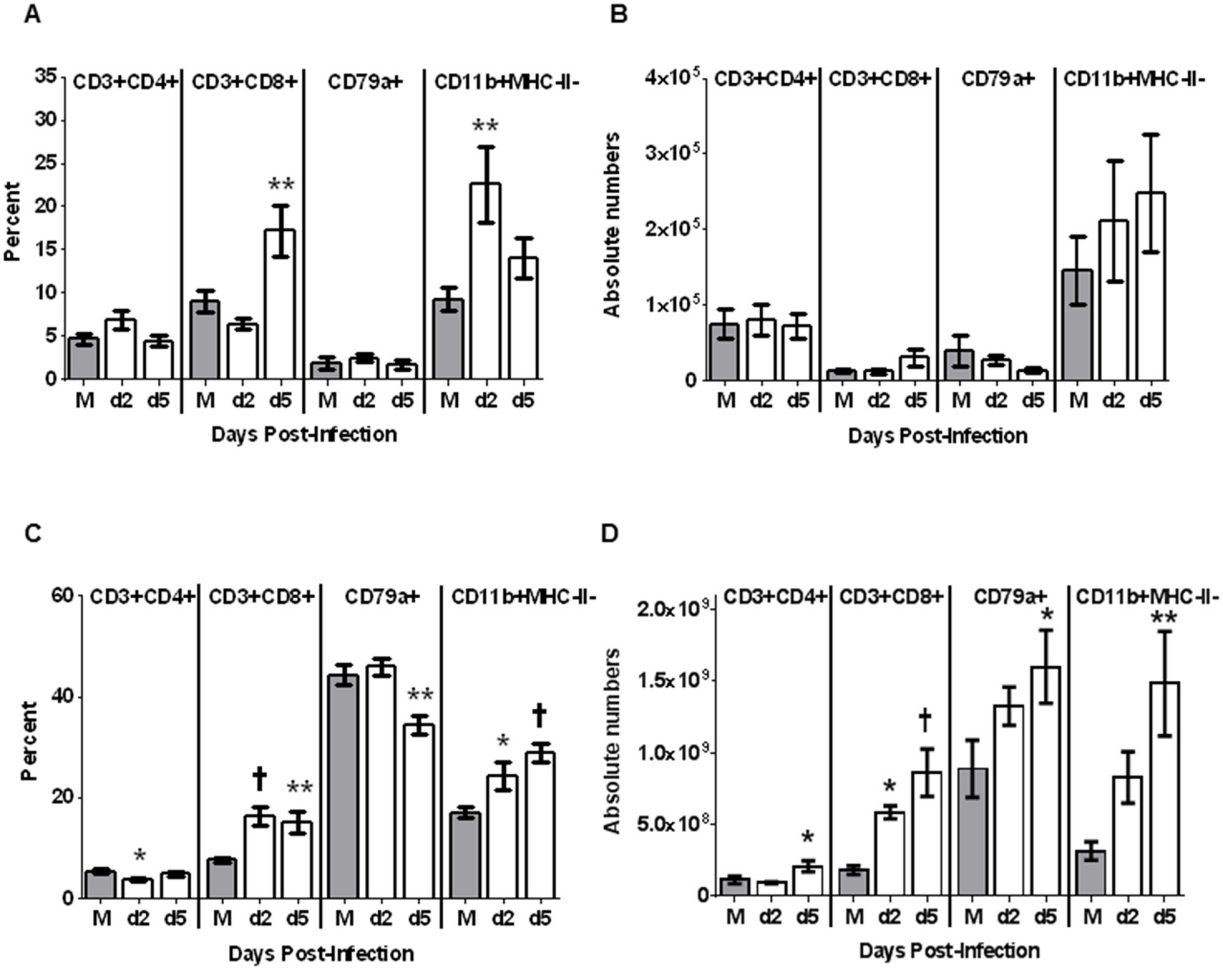
BALF and Spleen leukocyte subsets following infection. For screening of immune cell migration in response to influenza infection, BALF and Spleen were collected. Purified cell subsets from BALF (A-B) and Spleen (C-D) were stained and analyzed by flow cytometry. Percentages (A, C) and absolute number (B, D) of T_H_ (CD3+ and CD4+), Tc lymphocytes (CD3+ and CD8+), B cells (CD79a+) and CD11b-positive cells were measured. For mock infected animals (M), BALF and spleen were screened at day 5 post-challenge and for Perth/16 infected animals, at days 2 and 5 post-challenge. A p value of 0.05 was used as the cutoff for statistical significance (* p ≤ 0.05; ** p ≤ 0.01; † p ≤ 0.001). Error bars represent SEM.

In BALF, CD4+ T cell and B cell (CD79+) (Fig 5A and 5B) percentages and total number of cells were similar in mock-infected animals and infected animals on day 2 and day 5 post-infection. As a percentage of BALF cells, CD8+ cells increased on day 5 post-challenge (P≤0.01) (Fig 5A) and granulocytes increased on day 2 post-challenge (p≤0.01). However, these changes were not significantly different as absolute numbers (Fig 5B). A subset of CD11b+ cells expressing high levels of MHCII was observed in BALF. As a percent of total cells, this population was reduced in Perth/16 infected animals on days 2 (p≤0.001) and 5 (p≤0.05) post-challenge (S2A Fig), but the change as absolute number of cells did not reach significance (S2B Fig).

In contrast to BALF, the percentage of T cells in spleen and MRLN did change significantly following infection. The relative numbers of CD4+ cells decreased in spleen on day 2 post-challenge (p≤0.05), while CD8+ cells and granulocytes increased (Fig 5C) on days 2 and 5 post-challenge. However, since splenic cellularity increased after infection (Fig 3A), the absolute number of CD4+ cells, as well as CD8+ cells and granulocytes, showed significant increases (Fig 5D) after infection. Similarly, while the relative numbers of B cells decreased (Fig 5C) in spleens 5 days post-challenge (p≤0.01), the total number of B cells significantly increased (Fig 5D) on day 5 post-challenge (p≤0.01).

**Fig 6:**
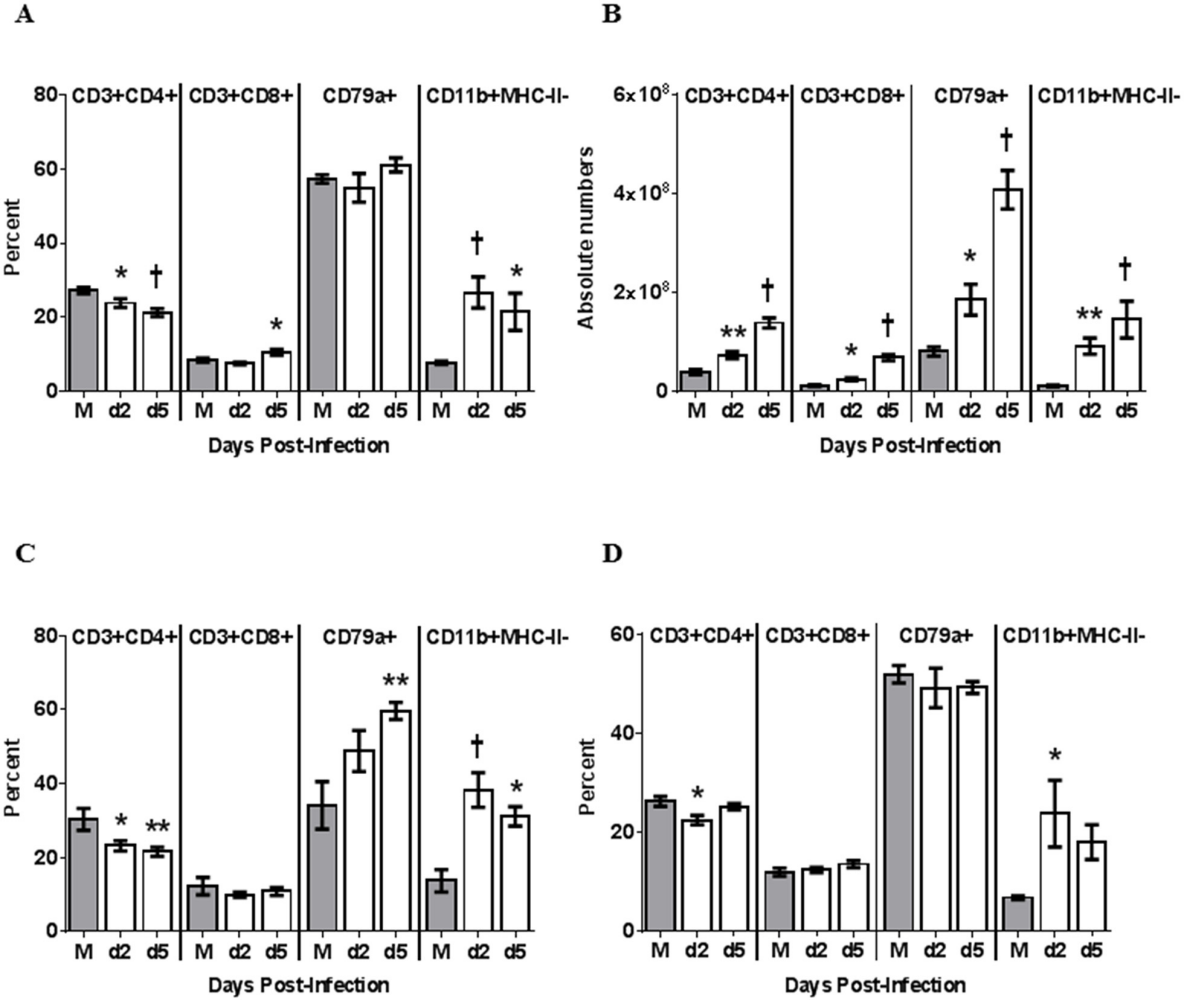
Lymph node leukocyte subsets following infection. For screening of immune cell migration in response to influenza infection, MRLN, MdLN and MsLN were collected. Purified cell subsets from MRLN (A-B), MdLN (C) and MsLN (D) were stained and analyzed by flow cytometry. In MRLN, percentage (A) and absolute number (B) of T_H_ (CD3+ and CD4+), Tc lymphocytes (CD3+ and CD8+), B cells (CD79a+) and CD11b-positive cells were measured. In MdLN and MsLN, only percentages (C-D) of T_H_ (CD3+ and CD4+), Tc lymphocytes (CD3+ and CD8+), B cells (CD79a+) and CD11b-positive cells were measured. For mock infected animals (M), LNs were screened at day 5 post-challenge and for Perth/16 infected animals at days 2 and 5 post-challenge. A p value of 0.05 was used as the cutoff for statistical significance (* p ≤ 0.05; ** p ≤ 0.01; † p ≤ 0.001). Error bars represent SEM.

We examined lymph nodes that were associated with the region of infection (MRLN and MdLN) or distant from the respiratory system (MsLN). When assessed as percentages, CD4+ T cells appeared to decrease in MRLN after infection, and B cells remained relatively constant (Fig 6A). However, as with splenocytes, the increasing cellularity of the MRLN (Fig 3A) was masking significant changes in absolute numbers of cells, since the total numbers CD4+ and CD8+ T cells, B cells, and granulocytes all increased in lymph nodes following infection (Fig 6B).

Although determining total cell numbers for the MdLN and MsLN was not possible due to their lack of clear demarcation, the pattern of relative cell numbers in these lymphoid tissues was roughly similar to that in MRLN. Both showed a similar relative reduction in CD4+ cells and an increase in granulocytes, however, MdLN alone demonstrated a relative increase in B cells (Fig 6C and 6D).

## Discussion

Human infection with influenza viruses leads to a wide range of disease severity, from subclinical to lethal disease [29]. In some cases, disease is associated with secondary bacterial infections [30–32], but in other cases influenza virus alone leads to localized or systemic inflammation and severe disease [29, 33]. Infection with highly pathogenic influenza such as H5N1 and H7N9 induces extensive infiltration of macrophages and neutrophils, upregulation of pro-inflammatory cytokines such as TNF-α, IL-1β, IL-6, IL-8 and IP-10, and cell death resulting in severe pulmonary inflammation [34–37]. Indeed, in animal models, influenza disease can be markedly reduced using anti-inflammatory therapeutics [9, 10, 38], suggesting that the inflammation itself is responsible for many of the symptoms of disease.

Mice are a convenient animal model [39]; they are easily housed and handled, and a large repertoire of mouse-specific reagents and transgenic and knock-out strains are available for analyzing host responses to infection or immunization. However, mice are not natural hosts for influenza virus, and human influenza viruses usually require adaptation to efficiently replicate and cause disease in mice [39–42]. The mouse model and mouse-adapted influenza strains may not accurately recapitulate natural infection of humans. Ferrets are considered the most appropriate model animal for the study of human influenza disease and immunity. They can be infected directly with human virus isolates and generally show similar clinical signs and disease severity to humans infected with similar viruses [12, 13].

We have previously used ferret-specific and cross-reactive antibodies to identify major leukocyte subsets, including CD4 and CD8 T cells, B cells, granulocytes, and cells of the monocyte lineage [18]. We found that following infection with seasonal strains of influenza (human A(H1N1)pdm09 and A(H3N2) strains), leukocytes in the peripheral blood showed dramatic changes in number even when the clinical signs associated with infection were minor. Lymphocytes, especially CD8+ T cells, were markedly though transiently depleted from the peripheral blood two days post-infection and gradually recovered over a period of about a week, while granulocytes showed a significant increase in peripheral blood counts on day two post-infection and also returned to normal over 5–7 days.

The changes in peripheral blood leukocyte numbers and ratios presumably reflect inflammation associated with influenza infection as well as the processes which keep inflammation in check. In particular, the depletion of lymphocytes in the first 3 days post-infection could reflect some combination of cell death due to apoptosis of lymphocytes sensitized by cytokines [43–45]; migration into the site of infection (i.e. the respiratory tract) [21]; or accumulation in secondary lymphoid organs [46–48]. In the present study, in order to better understand inflammation associated with influenza virus infection, we measured cell trafficking to various tissues following influenza virus infection of ferrets. We measured cell counts in the blood, BALF, and spleen, as well as in lymph nodes that were associated with the region of infection (MRLN and MdLN) or distant from the respiratory system (MsLN).

Seasonal influenza viruses, such as the one used in this study, induce mild clinical symptoms and have strong tropism for the upper respiratory tract, trachea, and bronchi [33, 49]. In uncomplicated seasonal influenza infections, early inflammatory responses are believed to help stimulate adaptive immunity in the form of B cells and T cells [50] which help eliminate virus, while a counter-acting anti-inflammatory response keeps inflammation in check. This orchestrated response typically results in viral clearance within a few days. In our study, dynamic changes in leukocytes in the lungs were obvious, although these data were quite variable (probably because of dynamic changes associated with an ongoing immune response at the site of infection as well as challenges associated with recovery of lavage fluid from lungs) and only a few changes were statistically significant (Fig 5). The draining lymph nodes, on the other hand, demonstrated highly orchestrated, less variable responses (Fig 6). All cell types assessed in this study accumulated significantly in MRLN by the 2^nd^ day post infection, further expanding by day 5 (Fig 6). Due to the diffuse nature of the MdLN, absolute numbers could not be calculated. However, as assessed by cell type percentages, MRLN and MdLN were very similar (Fig 6), suggesting that MRLN may be an effective surrogate for MdLN when assessing responses in the draining lymph nodes, with the added benefit of assessment of changes in absolute number of each cell type. Only B cells (CD79+) appear to vary between the two lymph nodes. It is possible that B cells preferentially accumulate in MdLN for the local production of antibody, due to their proximity to the lungs.

Granulocytes, composed primarily of neutrophils, are one of the first responders in an immune response. While once considered to function primarily as pro-inflammatory, phagocytic cells whose primary role was to limit infection until engagement of adaptive immunity, the role of neutrophils in protection from infection has more recently been found to be much more complex and diverse. While neutrophils have been shown to promote inflammation [51] and recruit pro-inflammatory macrophages [52], they also have been shown to play a role in resolution of inflammation toward the end of the inflammatory response [53]. Neutrophils furthermore have been shown to act as antigen presenting cells carrying engulfed antigen to the draining lymph nodes and supporting the CD8+ T cell response in the infected lung, possibly through antigen presentation at the site of infection [54, 55].

This population showed a dramatic increase on the second day post-infection (Fig 4) with the proportion of these cells significantly increased in the lungs (Fig 5), the site of infection. These cells also dispersed into the lymph nodes (Figs 5 and 6) showing dramatic accumulation not only in the draining lymph nodes (MRLN and MdLN) and spleen, but also in the more distant MsLN. Granulocytes were shown to further accumulate through at least day 5 post-infection in tissues for which we were able to assess absolute numbers; we were unable to determine absolute numbers of granulocytes in the more diffuse tissues, and changes were likely obscured due to the increased overall cellularity in these lymph nodes at day 5 of the ensuing immune response.

A population of CD11b+ cells expressing high levels of MHCII were observed in the lungs which decreased in response to influenza infection (S2A Fig). This population may represent lung antigen presenting cells, but confirmation and further characterization awaits more definitive cellular markers for ferret antigen-presenting cells.

T cell numbers dropped dramatically in the peripheral blood 2 days post-infection (Fig 4), accumulating in lymph nodes (Fig 6). CD8+ T cells also demonstrated significant accumulation in the spleen 2 days post-infection, in contrast to CD4+ T cells which were little changed (Fig 5). By day 5 post-infection, T cells further were more abundant in lymphoid organs, and CD8+ T cells became a much larger proportion of BALF cells at this point (Figs 5 and 6). The accumulation of lymphocytes in lymphoid tissues (especially lymph nodes) on day 2 probably represents mainly redistribution from the peripheral blood, since at this time point lymphocytes were severely depleted from the blood; by day 5, the large accumulation of lymphocytes in lymph nodes, with only moderate reduction in the blood, suggests that most of the cells in the lymph nodes were the result of expansion in situ.

CD8+ T cells function primarily through lysis of infected cells. These cells are activated in the lymph nodes, then traffic to the site of infection were they kill infected cells, thus hampering virus expansion. Although this process is necessary for the elimination of infection, the resulting tissue damage promotes inflammation. Furthermore, several of the granzymes, lytic mediators released by CD8+ T cells, have recently been associated with promotion of inflammation and induction of pro-inflammatory cytokines [56]. CD4+ T cells function primarily through production of cytokines to support and direct the ensuing immune response, although a subset of CD4+ T cells have been shown to have cytotoxic activity [57]. Both CD8+ and CD4+ T cells have been implicated in immunopathogenesis [58], but also function to keep inflammation in check to control immunopathology. In particular, both T cell subsets have been shown to be major producers of IL-10, a potent anti-inflammatory cytokine, during influenza virus infection [11, 59]. The relatively mild signs shown by ferrets infected with Perth/16 suggests that, in spite of the rapid accumulation and expansion of inflammatory cell types in and around the lungs, anti-inflammatory processes effectively kept inflammation in check while still allowing effective control of influenza infection.

These results clearly show dynamic migration and homing of lymphocytes in ferrets infected with seasonal human A influenza virus. Each of the four biological compartments examined responded differently to influenza infection. Understanding the dynamic patterns of immune cells trafficking within each compartment, how they are linked and how they are temporally and geographically specific, is critical to a systems biology understanding of the host immune response to influenza. Monitoring leukocyte homing and trafficking will aid in the determination of a role for cell-mediated immunity in protection from influenza as well as providing a more detailed view of the inflammatory impact of influenza virus infection.

## Supporting Information

S1 Fig Peripheral blood T cells following infection. Ferrets were bled on days 0–5 relative to the day of viral challenge, and cells were stained and analyzed by flow cytometry as described in the text. Percentage (A) and absolute number of T cells (CD3+) (B) were measured. For each animal the frequencies were normalized to the ferret’s values on day 0; the Y axis represents percent of values on day 0. Group averages are reported here. “Mock” ferrets were infected intranasally with sterile egg allantoic fluid; “Perth/16” ferrets infected intranasally with 1 x 10^6^ pfu of A/Perth/16/09. For mock animals (M), absolute number of same cell subsets were measured at day 5 post-challenge and for the infected animals at days 2 and 5 post-challenge. A p value of 0.05 was used as the cutoff for statistical significance (* p ≤ 0.05; ** p ≤ 0.01; † p ≤ 0.001). Error bars represent SEM.
(TIF)

S2 Fig BALF, Spleen and LN T cells and CD11b+MHCII+ cells following infection. For screening of immune cell migration in response to influenza infection, BALF, spleen, MRLN, MdLN and MsLN were collected. Purified cell subsets from BALF (A-B), spleen (C-D), MRLN (E-F), MdLN (G) and MsLN (H) were stained and analyzed by flow cytometry. In BALF, spleen and MRLN, percentage (A, C, E) and absolute number (B, D, F) of cells were measured. In MdLN and MsLN, only percentages (G, H) were measured. For the mock infected animals (M), tissues were screened at day 5 post-challenge and for the Perth/16 infected animals, at days 2 and 5 post-challenge. A p value of 0.05 was used as the cutoff for statistical significance (* p ≤ 0.05; ** p ≤ 0.01; † p ≤ 0.001). Error bars represent SEM.
(TIF)
