# Supplementary figures and images for: Peripheral Leukocyte Migration in Ferrets in Response to Infection with Seasonal Influenza Virus
Source: PLoS One. 2016 Jun 17;11(6):e0157903. doi: 10.1371/journal.pone.0157903 (PMC4912066; doi:10.1371/journal.pone.0157903)

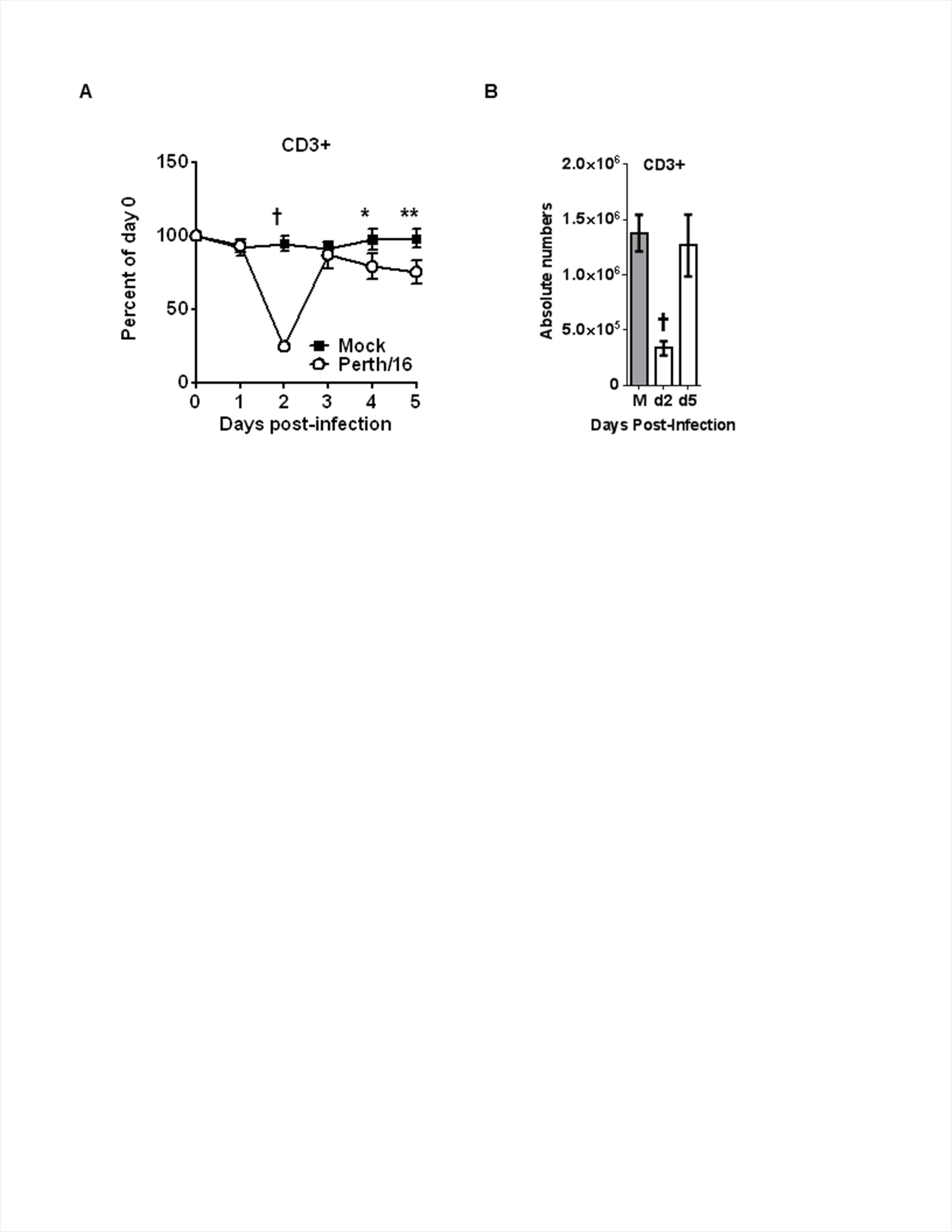

Supplement: S1 Fig — Ferrets were bled on days 0–5 relative to the day of viral challenge, and cells were stained and analyzed by flow cytometry as described in the text. Percentage (A) and absolute number of T cells (CD3+) (B) were measured. For each animal the frequencies were normalized to the ferret’s values on day 0; the Y axis represents percent of values on day 0. Group averages are reported here. “Mock” ferrets were infected intranasally with sterile egg allantoic fluid; “Perth/16” ferrets infected intranasally with 1 x 10^6^ pfu of A/Perth/16/09. For mock animals (M), absolute number of same cell subsets were measured at day 5 post-challenge and for the infected animals at days 2 and 5 post-challenge. A p value of 0.05 was used as the cutoff for statistical significance (* p ≤ 0.05; ** p ≤ 0.01; † p ≤ 0.001). Error bars represent SEM. (TIF) [file pone.0157903.s001.tif]

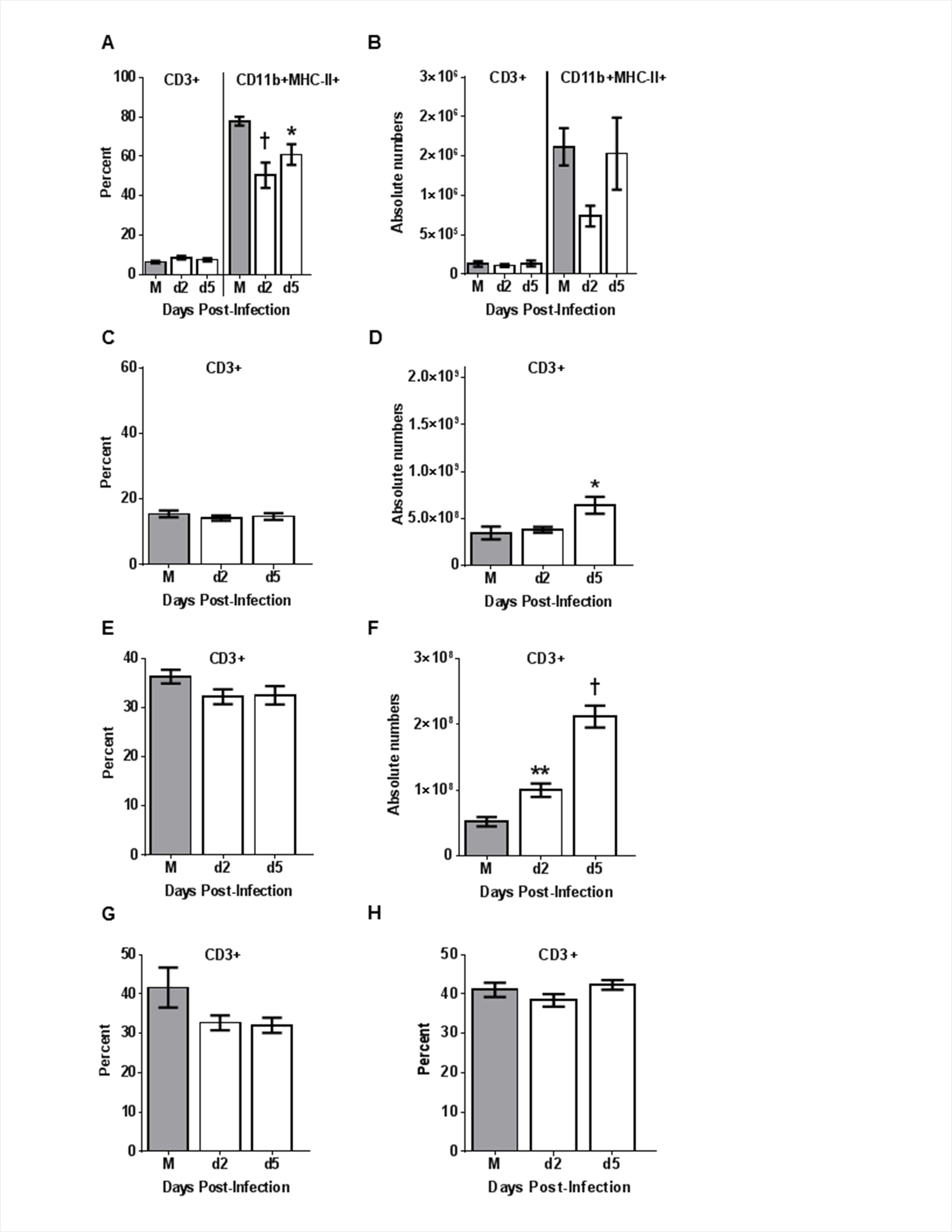

Supplement: S2 Fig — For screening of immune cell migration in response to influenza infection, BALF, spleen, MRLN, MdLN and MsLN were collected. Purified cell subsets from BALF (A-B), spleen (C-D), MRLN (E-F), MdLN (G) and MsLN (H) were stained and analyzed by flow cytometry. In BALF, spleen and MRLN, percentage (A, C, E) and absolute number (B, D, F) of cells were measured. In MdLN and MsLN, only percentages (G, H) were measured. For the mock infected animals (M), tissues were screened at day 5 post-challenge and for the Perth/16 infected animals, at days 2 and 5 post-challenge. A p value of 0.05 was used as the cutoff for statistical significance (* p ≤ 0.05; ** p ≤ 0.01; † p ≤ 0.001). Error bars represent SEM. (TIF) [file pone.0157903.s002.tif]
